# Supplementary material for: Steroid profiling in human primary teeth via liquid chromatography-tandem mass spectrometry for long-term retrospective steroid measurement
Source: PLoS One. 2024 Aug 28;19(8):e0309478. doi: 10.1371/journal.pone.0309478 (PMC11357110; doi:10.1371/journal.pone.0309478)
Supplement: Attachment — Submitted filename: 17Jun2024_Response to Reviewers.docx [file pone.0309478.s001.docx]

**Manuscript Number: PONE-D-24-09259**

**We thank the reviewers for their helpful comments and suggestions that allowed us to greatly improve this manuscript. We have addressed every comment point by point, as detailed below (changes in the revised manuscript are in blue). Our revisions focused on clarifying methodologies for steroid extraction and steroid quantification by LC-MS/MS. In particular, we have revised Table 3 to include LLOQs and LODs and revised Figure 2. Additional details about our methods and participants have been provided in the Methods section. We also deposited our laboratory protocols in protocols.io (DOI:** **dx.doi.org/10.17504/protocols.io.e6nvw1b3dlmk/v1). Lastly, we revised the section of the Discussion about testosterone levels.**

Reviewer #1:

Reviewer #1: The manuscript is well written and the method is extensively described. I have, however, a few unclear issues.

1. Was the use of polypropylene tubes unavoidable in the extraction procedure? I am asking this since plastic contaminants can increase background in MS/MS. Did blank samples go through the same extraction procedures as the tooth powder?

**Response: We avoided using plastics, such as polypropylene, in the extraction procedure as much as possible. However, tubes for the homogenization are made from polypropylene. Also, for the final step of the extraction process, we centrifuge at a high speed (over 16,000× *g*) and this requires polypropylene microcentrifuge tubes. However, the contact time of samples with these microcentrifuge tubes is minimized (~7 min total). We agree with the reviewer that plastic contaminants can increase background; however, our method showed high selectivity for the target analytes, as double blank samples did not contain any interferences in the monitored analytes and internal standard MRM transitions.**

**Yes, blanks and double blanks went through the same extraction procedure as the tooth samples, and no signals were observed (Fig. 1).**

2. At what point were the internal standards added to the sample or blank? Did the internal standards also go through the extraction procedure?

**Response: The internal standards were added to the calibration curves, quality controls, blanks, and samples (but not the double blanks) prior to homogenization and steroid extraction. Therefore, the internal standards went through the entire extraction procedure. We have now clarified this point in the Methods section (line 176 and lines 179-181).**

**“Prior to homogenization, 50 μl of deuterated** **internal standards (dehydroepiandrosterone-d6, testosterone-d5, cortisol-d4, corticosterone-d8, progesterone-d9, pregnenolone-d4,** **allopregnanolone-d4, 17β-estradiol-d4, and aldosterone-d7 C/D/N Isotopes Inc., Pointe-Claire, Canada) in 50:50 HPLC-grade methanol:MilliQ water were added to the calibration curves, quality controls, blanks, and samples (40 pg/sample of DHEA-d6, pregnenolone-d4, allopregnanolone-d4, and 20 pg/sample of all other internals standards).”**

3. What were the LOQ for the analytes of interest?

**Response: The LLOQs were the lowest concentration on the calibration curve (2.0** **pg/10 μl for DHEA, pregnenolone, and allopregnanolone, and 0.05 pg/10 μl for all other steroids) within 20% of the nominal concentration.**

**We have now revised Table 3 to include LLOQs.**

**“Table 3 Coefficient of determination (*r*^2^), p-value and lower limit of quantification (LLOQ) of LC-MS/MS calibration curves.**

| **Steroid** | ***r*^2^** | ***p*** | **LLOQ (pg/10 μl)** | **Intra-assay %CV** | **Inter-assay %CV** | |
| --- | --- | --- | --- | --- | --- | --- |
| **DHEA** | **0.9946** | **<0.0001** | **2.0** | **1.0** | **4.8** |  |
| **Androstenedione** | **0.9912** | **<0.0001** | **0.05** | **2.4** | **3.2** |  |
| **5α-Dihydrotestosterone** | **0.9967** | **<0.0001** | **0.05** | **0.3** | **4.7** |  |
| **Testosterone** | **0.9991** | **<0.0001** | **0.05** | **0.3** | **5.1** |  |
| **Cortisone** | **0.9959** | **<0.0001** | **0.05** | **1.1** | **3.1** |  |
| **Cortisol** | **0.9976** | **<0.0001** | **0.05** | **5.8** | **6.9** |  |
| **11-Deoxycortisol** | **0.9969** | **<0.0001** | **0.05** | **0.7** | **3.4** |  |
| **11-Deoxycorticosterone** | **0.9982** | **<0.0001** | **0.05** | **0.6** | **5.4** |  |
| **DHC** | **0.9962** | **<0.0001** | **0.05** | **3.9** | **4.5** |  |
| **Corticosterone** | **0.9993** | **<0.0001** | **0.05** | **2.1** | **4.2** |  |
| **Progesterone** | **0.9975** | **<0.0001** | **0.05** | **2.0** | **0.3** |  |
| **Pregnenolone** | **0.9987** | **<0.0001** | **2.0** | **5.9** | **7.2** |  |
| **Allopregnanolone** | **0.9906** | **<0.0001** | **2.0** | **3.7** | **4.6** |  |
| **Estrone** | **0.9966** | **<0.0001** | **0.05** | **3.0** | **5.4** |  |
| **Estriol** | **0.9988** | **<0.0001** | **0.05** | **5.0** | **5.5** |  |
| **17β-estradiol** | **0.9978** | **<0.0001** | **0.05** | **1.3** | **4.4** |  |
| **Aldosterone** | **0.9980** | **<0.0001** | **0.05** | **2.8** | **3.1** |  |

**Note: The limit of detection (LOD) for aldosterone, DHC, cortisol, and corticosterone was 0.025 pg/10 μl. The LODs for progesterone, pregnenolone, and DHEA were 0.01 pg/10 μl, 0.015 pg/10 μl, and 0.2 pg/10 μl, respectively.”**

4. What was the volume for the calibration curves? You mention that the standard curve started at 0.05 pg, this seems extremely low, what was the minimal concentration used?

**Response: For each point on the calibration curves, we used 10** **μl of steroid in 50:50 HPLC-grade methanol:MilliQ water. The concentrations of the standards ranged from 0.005 pg/μl to 100 pg/μl. Therefore, the calibration curve ranged from 0.05 to 1000 pg per tube. We agree that the LLOQs are extremely low, making the method very sensitive.**

5. What was the number of replicates for intra-assay CV analysis? What about the inter-assay CV, how many independent analyses were included in the calculation? Same question for recovery analysis and linearity.

**Response:** **Intra- and inter-assay quality controls were assessed in triplicate for each run. Inter-assay variation (%CV) was calculated from the results from 4 independent assays. To assess recovery, extracts from 6 teeth were pooled and split into 2 groups, either spiked with known amounts of steroid or unspiked, with 5 replicates per group. Linearity was assessed in singleton.**

6. Why are there higher levels of Testosterone for female samples? Testosterone is still lower even in infant girls than boys. Can the higher levels of Testo in female primary teeth be explained? You mention that male fetuses are exposed to surging levels of Testo for several weeks, how are they still lower than female levels?

**Response: Statistical analyses showed no significant difference in testosterone between males and females (*p* = 0.29). The non-significant trend for higher testosterone levels in female samples than in male samples was unexpected to us. However, at this time, we conclude that there was no significant difference in testosterone levels in primary teeth from males and females. Future studies may examine more samples to see if this pattern is replicable.**

**We have revised the Discussion (lines 395-397).**

**“No significant sex differences in steroids were detected in the primary bottom central incisors. If steroids are deposited during tooth formation (from week 6 of gestation to 18-36 months after birth) then primary teeth reflect steroid levels before puberty, which may account for the lack of sex differences [28-31****]. Surprisingly, testosterone showed a suggestion to be higher in females than in males, but this was not statistically significant. More samples should be examined to see if this pattern is replicable.”**

7. Minor comment: on page 14 line 247 I think is meant to cite Table 3.

**Response: This has been corrected in the Results section (line 254-255).**

**“Intra-assay and inter-assay variation (% CV) were below 5%, confirming the precision of the assay (Table 3).”**

Reviewer #2

Reviewer #2: General Comments
I find the manuscript to be of interest to other researchers in the field. The paper presents a new method to quantify steroid level in human primary teeth. The study is interesting and easy to read. The authors must please report the method validation according to analytical chemistry specifications (see specific comments). I am also concerned that the reported values where calculated correctly (see specific comments)
If the authors can please just check and correct the calculations, the manuscript can be accepted for publication.

Headings should be numbered.

**Response: The headings were formatted to follow PLOS ONE's style requirements, as per the instructions to authors on the journal website. Thus, instead of numbering, we have formatted them into level 1, 2, and 3 headings, in bold type 18pt, 16pt, and 14pt font, respectively.**

Introduction:
Line 67
…primary teeth’s chemical composition.

**Response: We have corrected this in the Introduction section (line 67).**

**“Environmental exposures are also reflected in primary teeth’s chemical composition [27, 28, 35, 36].”**

Development and Validation
Line 91 – please add the age range.

**Response: We added this information to the Development and Validation section (line 92-93).**

**“Human primary teeth were from male and female participants ranging from 4 to 8 years of age.”**

Table 1 – what significance level are you using for your p value?

**Response: Significance criterion was set at *p* ≤ 0.05. We have now clarified this as a note in Table 1.**

**“Note: Data are shown as mean ± *SEM. n* = 8 males and 9 females. Significance criterion was set at *p* ≤ 0.05.”**

Steroid Analysis by LC-MS/MS
Line 143 - 45 ul is a quite a large injection volume for an UPLC, especially considering you reconstituted in 55 ul and then also only transferred the supernatant to the LC vials. I am not sure if you were able to inject all 45 ul. Did you check and adjust the autosampler needle?

**Response: The volume of the pellet was negligible; therefore, nearly all of the 55 μl was transferred and available for injection (50-55 μl). The volume remaining after injection was assessed and shown to be 5-10 μl.**

**We are completely confident that we were able to inject all 45 μl here and in our previous papers [1,2].**

Line 153 – the needle wash on these instruments are usually a strong and weak wash. Just check the method.

**Response: The instrument setting was optimized for needle wash using 100% methanol. This wash was effective in preventing carryover (i.e. cross-contamination between samples). Neat solvents run after needle wash did not contain MRM transitions matching with the analytes and internal standards.**

Line 164 – please give calibration range, ie what is the volulme of the tube (pg per tube is not meaningful to readers).

**Response: The volume of the calibration curves was 10 μl. The concentration of the standards ranged from 0.005 pg/μl to 100 pg/μl. Therefore, the calibration curve ranged from 0.05 to 1000 pg per tube.**

Line 195 – what is meant by “vehicle”?

**Response: We have removed the term vehicle from the manuscript (line 198).**

**“The tubes were split into two groups (n = 6/group) and spiked with either known amounts of steroid (based on expected steroid concentrations; 50 pg of DHEA, 5 pg of cortisol, and 2 pg of all other steroids) or unspiked.”**

Results
Please check figure 2 – calibration curves should not go through 0 (I can’t clearly tell from the image). Please add line of fit equation on the figures.

The reporting of the calibration curve ranges are confusing.

**Response: We have now revised Figure 2. The point at the origin has been removed, and the line of fit equations of the calibration curves have been added to the legend.**

**“Fig 2. Calibration curves for the 3 most abundant steroids in human primary teeth (A) DHEA, (B) cortisol, and (C) progesterone. Area ratio is calculated by dividing the peak area of the analyte by the peak area of the corresponding deuterated internal standard in the same sample. Calibration curve range was 2 to 1,000 pg per tube for DHEA and 0.05 to 1,000 pg per tube for cortisol and progesterone. Insets display the lowest standards on the calibration curves and demonstrate the excellent assay sensitivity.** **The line of fit equations are y = 0.0477*x* + 0.0802, y = 0.0650*x* + 0.1729, y = 0.0039*x* + 0.0250 for DHEA, cortisol, and progesterone, respectively.”**

Lines 226 – 229 – the range is 0.05 to 1000 pg or 2 to 1000 pg depending on the steroids. However, in Fig 2’s legend you are reporting 2 to 1000 or 0.05 to 1000 pg per tube.

**Response: Steroids such as DHEA, pregnenolone, and allopregnanolone, show lower ionization efficiencies, and thus, assay sensitivity is lower. Instead of a lower limit of quantification at 0.05 pg, their lower limit of quantification is at 2 pg. Therefore, in Figure 2, DHEA is reported to range from 2 to 1000 pg per tube, while cortisol and progesterone range from 0.05 to 1000 pg per tube.**

The authors mention gram amounts, I am concerned that the gram amount was directly read off the calibration curve for the samples. This is not correct as the final sample volume and the standards’ tube volume (from what I can tell) is not the same. Please confirm that the correct calculations were done.

**Response: In this manuscript, we reported the level of steroids in teeth as ng/g or pg/tooth. The raw data were obtained in pg, using a calibration curve that ranged from 0.05 to 1000 pg per tube. The concentrations of the standards ranged from 0.005 pg/μl to 100 pg/μl. For each point on the calibration curves, we used 10 μl of steroid in 50:50 HPLC-grade methanol:MilliQ water. For each tube, steroids from an entire tooth were assessed. The obtained quantitative value in pg was then converted to ng/g, based on the weight of each tooth. For steroid analysis in teeth, final results in ng/g are more suitable and useful as samples are in solid form. In our previous work, as well as other various published work on tissues, data are reported in ng/g [1,2].**

Method validation (for analytical methods) should be reported as %RSD, recovery, LODs and LOQs for all analytes, please add.

**Response: We have revised Table 3 to include the LLOQs and LODs in the note below the table. Inter- and intra- assay %CVs (%RSD) were already reported in Table 3, and recovery has already been reported in Table 5.**

**“Table 3 Coefficient of determination (*r*^2^), p-value, and lower limit of quantification (LLOQ) of LC-MS/MS calibration curves.**

| **Steroid** | ***r*^2^** | ***p*** | **LLOQ (pg/10 μl)** | **Intra-assay %CV** | **Inter-assay %CV** | |
| --- | --- | --- | --- | --- | --- | --- |
| **DHEA** | **0.9946** | **<0.0001** | **2.0** | **1.0** | **4.8** |  |
| **Androstenedione** | **0.9912** | **<0.0001** | **0.05** | **2.4** | **3.2** |  |
| **5α-Dihydrotestosterone** | **0.9967** | **<0.0001** | **0.05** | **0.3** | **4.7** |  |
| **Testosterone** | **0.9991** | **<0.0001** | **0.05** | **0.3** | **5.1** |  |
| **Cortisone** | **0.9959** | **<0.0001** | **0.05** | **1.1** | **3.1** |  |
| **Cortisol** | **0.9976** | **<0.0001** | **0.05** | **5.8** | **6.9** |  |
| **11-Deoxycortisol** | **0.9969** | **<0.0001** | **0.05** | **0.7** | **3.4** |  |
| **11-Deoxycorticosterone** | **0.9982** | **<0.0001** | **0.05** | **0.6** | **5.4** |  |
| **DHC** | **0.9962** | **<0.0001** | **0.05** | **3.9** | **4.5** |  |
| **Corticosterone** | **0.9993** | **<0.0001** | **0.05** | **2.1** | **4.2** |  |
| **Progesterone** | **0.9975** | **<0.0001** | **0.05** | **2.0** | **0.3** |  |
| **Pregnenolone** | **0.9987** | **<0.0001** | **2.0** | **5.9** | **7.2** |  |
| **Allopregnanolone** | **0.9906** | **<0.0001** | **2.0** | **3.7** | **4.6** |  |
| **Estrone** | **0.9966** | **<0.0001** | **0.05** | **3.0** | **5.4** |  |
| **Estriol** | **0.9988** | **<0.0001** | **0.05** | **5.0** | **5.5** |  |
| **17β-estradiol** | **0.9978** | **<0.0001** | **0.05** | **1.3** | **4.4** |  |
| **Aldosterone** | **0.9980** | **<0.0001** | **0.05** | **2.8** | **3.1** |  |

**Note: The limit of detection (LOD) for aldosterone, DHC, cortisol, and corticosterone was 0.025 pg/10 μl. The LODs for progesterone, pregnenolone, and DHEA were 0.01 pg/10 μl, 0.015 pg/10 μl, and 0.2 pg/10 μl, respectively.”**

SEX DIFFERENCES
Line 297 – please define “GCs”

**Response: This has now been defined in the Results section (line 305).**

**“All glucocorticoids (GCs) were significantly positively correlated with each other when both sexes were combined.”**

Discussion

Please add is the levels detected of concern. Why is some levels higher for some teeth?

**Response: This comment is unclear to us, but we believe it may be asking about the clinical significance of the dental steroid levels, and whether these differences can be explained.**

**Our study is the first to examine multiple steroids in human primary teeth by LC-MS/MS, so the clinical significance awaits further study. Since various organic and inorganic compounds have been detected in primary teeth, it is hypothesized that circulating steroids may be deposited during tooth formation, which begins *in utero*. Thus, elevated steroids may reflect pathologies involving the dysregulation of steroid hormone secretion during perinatal life.**

**We discuss this point in paragraphs 3-4 of the Introduction (lines 60-77). We also suggest** **studies in the Discussion to further investigate this hypothesis. Specifically, correlation studies between steroid levels in primary teeth and developmental stressors such as maternal illness or low socioeconomic status (lines 401-403).**

References

1. Hamden JE, Gray KM, Salehzadeh M, Kachkovski GV, Forys BJ, Ma C, Austin SH, Soma KK. Steroid profiling of glucocorticoids in microdissected mouse brain across development. Dev Neurobiol. 2021;81(2):189-206. Epub 2021/01/10. doi: 10.1002/dneu.22808. PubMed PMID: 33420760.
2. Jalabert C, Ma C, Soma KK. Profiling of systemic and brain steroids in male songbirds: Seasonal changes in neurosteroids. J Neuroendocrinol. 2021 Jan;33(1):e12922. doi: 10.1111/jne.12922. Epub 2020 Dec 14. PMID: 33314446.
