# Supplementary material for: Steroid profiling in human primary teeth via liquid chromatography-tandem mass spectrometry for long-term retrospective steroid measurement
Source: PLoS One. 2024 Aug 28;19(8):e0309478. doi: 10.1371/journal.pone.0309478 (PMC11357110; doi:10.1371/journal.pone.0309478)
Supplement: Attachment — Submitted filename: 11Jul2024_Response to Reviewers.docx [file pone.0309478.s003.docx]

**Manuscript Number: PONE-D-24-09259R1**

**We thank Reviewer #2 for their helpful comments and suggestions. We have addressed every comment point by point, as detailed below (changes in the revised manuscript are in blue). Our revisions focused on clarifying the calibration curve and the calculation of sample concentrations. In particular, Table 3 has been revised further to clarify how we determined the LLOQs and LODs. Additional details have been provided in the Methods and Results sections.**

Reviewer #2

Steroid Analysis by LC-MS/MS

(please note for future analysis it is recommended to use MS grade solvents and not HPLC grade

for mass spec)

**Response: Thank you for this suggestion. We have compared MS-grade and HPLC-grade solvents in the past but will revisit this point in the future, if necessary.**

Line 169 – Please add the amount of liquid added to the standard. – the 10ul.

**Response:**

**The *initial* amount of liquid added to the standards (10 μl) has been added to the Methods section (line 166-174). Note that these standards in the calibration curve are processed in the same way as the unknowns (i.e. teeth samples) and resuspended in 55 μl prior to injection. This important point is now clearer in the Methods.**

**“****Standards ranged from 0.005 to 100 pg/μl. 10 μl of each standard was used to make the calibration curves. The calibration curves were then processed alongside the samples under the same extraction procedure. Both the calibration curves and the teeth samples were dried in a vacuum centrifuge and resuspended in the same final volume of 55 μl of 1:3 HPLC-grade methanol:MilliQ water. The calibration curve range was 0.05 to 1000 pg per tube (0.91 to 18,182 pg/ml) for all steroids (excluding DHEA, pregnenolone, and allopregnanolone) and prepared in 50:50 HPLC-grade methanol:MilliQ water. The calibration curve range was 2 to 1000 pg per tube (36.4 to 18,182 pg/ml) for DHEA, pregnenolone, and allopregnanolone.”**

Also, note you have now reconstituted your teeth samples in 55ul and the standards in 10ul, thus it is not directly comparable.

For example, if for a sample you read off the calibration curve you detected 2ng that is 2ng/10ul = 0.2ng/ul.

Your sample was reconstituted into 55ul

So, 0.2ng/ul = 11ng/55ul

Thus, you had 11 ng in the original sample.

This you can then report per the gram of sample weighed.

**Response: We have revised the Methods to make it clearer that teeth samples and standards were all reconstituted in 55 μl (line 166-174). We have used similar protocols in many previous papers, including our most recent paper in *Nature* [1]. We apologize if this detail was unclear in the previous version of the manuscript.**

Also, what was the injection volume for the standards as it can not be 45ul seeing you made them in 10ul?

**Response: Both the standards and the teeth samples were reconstituted in the same volume of 55 μl. 10 μl is the *initial* volume of standard added to the tube. Standards were then processed alongside the teeth samples using the same extraction process. Therefore, both the standards and the teeth samples were treated the same way – dried in a vacuum centrifuge and resuspended in 55 μl of 1:3 HPLC-grade methanol:MilliQ water. The injection volume was 45 μl for both the standards and the teeth samples.**

**We have used this method in numerous published studies [1-5]. In previous studies and the present study, the quality controls and recovery values were as expected. We hope these points reassure the reviewer with regard to our calculations.**

Line 176 – Add prior to homogenization and extraction the IS was added…

**Response: We have revised this in the Methods section (line 182).**

**“Prior to homogenization and extraction, 50 μl of deuterated** **internal standards (dehydroepiandrosterone-d6, testosterone-d5, cortisol-d4, corticosterone-d8, progesterone-d9, pregnenolone-d4,** **allopregnanolone-d4, 17β-estradiol-d4, and aldosterone-d7 C/D/N Isotopes Inc., Pointe-Claire, Canada) in 50:50 HPLC-grade methanol:MilliQ water were added to the calibration curves, quality controls, blanks, and samples (40 pg/sample of DHEA-d6, pregnenolone-d4, allopregnanolone-d4, and 20 pg/sample of all other internals standards).”**

Results

Lines 232 – the calibration range is not 2 to 1000 pg as you could not possible inject all the standard (ie the entire for example 2ng on the column). Please report the calibration range as a

concentration. Also add the injection amount to give the gram on column per injection.

Fig 2’s legend you are reporting 2 to 1000 or 0.05 to 1000 pg per tube please report per 10ul.

**Response: We have revised the Results section to include the concentration of the calibration curve range and the injection amount (line 235-242).**

**“The calibration curve consisted of 13 points ranging** **from 0.05 to 1000 pg per tube (0.91 to 18,182 pg/ml) for androstenedione, 5α-dihydrotestosterone, testosterone, 11-deoxycortisol, cortisol, cortisone, 11-deoxycorticosterone, corticosterone, DHC, progesterone, estrone, 17β-estradiol, estriol and aldosterone. The calibration curve ranged from 2 to 1000 pg per tube (36.4 to 18,182 pg/ml) for DHEA, pregnenolone, and allopregnanolone. The on-column amount ranged from 0.041 pg to 818.2 pg per injection for all steroids (except DHEA, pregnenolone, and allopregnanolone). The on-column amount ranged from 1.64 pg to 818.2 pg per injection for DHEA, pregnenolone, and allopregnanolone. All steroids showed excellent linearity and displayed a coefficient of determination (r^2^) greater than 0.99 and *p*-values less than 0.0001 (Table 3) (Fig 2).”**

**While 10 μl of each standard was added to the calibration curves, these standards were then processed in the same way as the teeth samples and resuspended in 55 μl. Thus, we have revised Figure 2 to include the concentration in pg/ml instead (line 258-259).**

**“Calibration curve range was 2 to 1000 pg per tube** **(0.91 to 18,182 pg/ml) for DHEA and 0.05 to 1000 pg per tube** **(36.4 to 18,182 pg/ml) for cortisol and progesterone.”**

How LODs and LOQs were determined is not based on any standard methods. Please recalculate, you can use the 3S/N for LODs and 10S/N method for LOQs. You need to add to your method how it was determined.

**Response: The determination of the Limit of Detection (LOD) and the Lower Limit of Quantitation (LLOQ) was based on the FDA guidelines [6]. The LOD was selected as the lowest concentration at which the analyte produced a signal-to-noise (S/N) ratio ≥ 3. The LLOQ was selected as the lowest concentration with a S/N ratio ≥ 5, with an accuracy within ±20% of the nominal value and a coefficient of variation (CV) within ±20%.**

**We have added the LOD and LLOQ determination criteria to Table 3 (line 250-253).**

**“Note: The limit of detection (LOD) for aldosterone, DHC, cortisol, and corticosterone was 0.025 pg/10 μl. The LODs for progesterone, pregnenolone, and DHEA were 0.01 pg/10 μl, 0.015 pg/10 μl, and 0.2 pg/10 μl, respectively. The LOD was selected as the lowest concentration at which the analyte produced a signal-to-noise (S/N) ratio ≥ 3. The LLOQ was selected as the lowest concentration with a S/N ratio ≥ 5, with an accuracy within ±20% of the nominal value and a coefficient of variation (CV) within ±20% [51].”**

References

1. Niepoth, N., Merritt, J. R., Uminski, M., Lei, E., Esquibies, V. S., Bando, I. B., Hernandez, K., Gebhardt, C., Wacker, S. A., Lutzu, S., Poudel, A., Soma, K. K., Rudolph, S., & Bendesky, A. Evolution of a novel adrenal cell type that promotes parental care. *Nature*. 2024;629(8014):1082-1090. doi:10.1038/s41586-024-07423-y
2. Hamden JE, Gray KM, Salehzadeh M, Kachkovski GV, Forys BJ, Ma C, Austin SH, Soma KK. Steroid profiling of glucocorticoids in microdissected mouse brain across development. Dev Neurobiol. 2021;81(2):189-206. Epub 2021/01/10. doi: 10.1002/dneu.22808. PubMed PMID: 33420760.
3. Jalabert C, Ma C, Soma KK. Profiling of systemic and brain steroids in male songbirds: Seasonal changes in neurosteroids. *J Neuroendocrinol*. 2021;33(1):e12922. doi:10.1111/jne.12922
4. Hamden JE, Gray KM, Salehzadeh M, Soma KK. Isoflurane stress induces region-specific glucocorticoid levels in neonatal mouse brain. *J Endocrinol*. 2022;255(2):61-74. Published 2022 Sep 14. doi:10.1530/JOE-22-0049
5. Salehzadeh M, Hamden JE, Li MX, Bajaj H, Wu RS, Soma KK. Glucocorticoid Production in Lymphoid Organs: Acute Effects of Lipopolysaccharide in Neonatal and Adult Mice. Endocrinology. 2022;163(2):bqab244. doi:10.1210/endocr/bqab244
6. Food and Drug Administration. Bioanalytical Method Validation Guidance for Industry. 2018. [Cited 2024 July 11]. Available from: https://www.fda.gov/drugs/guidance-compliance-regulatory-information/guidances-drugs
