# Supplementary material for: Steroid profiling in human primary teeth via liquid chromatography-tandem mass spectrometry for long-term retrospective steroid measurement
Source: PLoS One. 2024 Aug 28;19(8):e0309478. doi: 10.1371/journal.pone.0309478 (PMC11357110; doi:10.1371/journal.pone.0309478)
Supplement: Attachment — Submitted filename: PONE-D-24-09259R1 Reviewer Comments.pdf [file pone.0309478.s002.pdf]

General Comments

I find the manuscript to be of interest to other researchers in the field. The paper presents a new method to quantify steroid level in human primary teeth. The study is interesting and easy to read.

The authors have still not provided the calculations to determine the concentrations of the targets in the tooth samples. There is not enough information given in the paper for me to check if the calculations are correct. With the information given I am struggling to agree with the concentration values reported.

I have given an example calculation for the authors to check if that is how they calculated the amount of target analytes.

Specific feedback to the authors is given here, if they wish to improve the manuscript:

Steroid Analysis by LC-MS/MS

(please note for future analysis it is recommended to use MS grade solvents and not HPLC grade for mass spec)

Line 169 – Please add the amount of liquid added to the standard. – the 10ul.

Also, note you have now reconstituted your teeth samples in 55ul and the standards in 10ul, thus it is not directly comparable.

For example, if for a sample you read off the calibration curve you detected 2ng that is 2ng/10ul = 0.2ng/ul.

Your sample was reconstituted into 55ul

So, 0.2ng/ul = 11ng/55ul

Thus, you had 11 ng in the original sample.

This you can then report per the gram of sample weighed.

Also, what was the injection volume for the standards as it can not be 45ul seeing you made them in 10ul?

Line 176 – Add prior to homogenization and extraction the IS was added…

Results

Lines 232 – the calibration range is not 2 to 1000 pg as you could not possible inject all the standard (ie the entire for example 2ng on the column). Please report the calibration range as a concentration. Also add the injection amount to give the gram on column per injection.

Fig 2's legend you are reporting 2 to 1000 or 0.05 to 1000 pg **per tube please report per 10ul.**

How LODs and LOQs were determined is not based on any standard methods. Please recalculate, you can use the 3S/N for LODs and 10S/N method for LOQs. You need to add to your method how it was determined.
